# 

**Published:** 1933

**Authors:** 


### CONTENTS

| | PAGE |
|---|---|
| FOREWORD . . . . . . . . . | 3 |
| EDITORIAL STAFF . . . . . . . | 4 |
| PRESIDENTS AND PRESIDENTIAL ADDRESSES . | 5 |
| THE LONG FOX MEMORIAL LECTURES . . | 9 |
| INDEX TO AUTHORS . . . . . . | 11 |
| INDEX TO SUBJECTS . . . . . . | 33 |
| OBITUARIES . . . . . . . . | 56 |

